# A rare case of Sporadic Creutzfeldt-Jakob disease at a remote mountain hospital in the Indian Himalayan Region

**DOI:** 10.4322/acr.2024.502

**Published:** 2024-06-21

**Authors:** Nitu Sharma, Jitender Kumar Sharma, Ashima Chander, Khushdeep Shergill, Meghna Yadav

**Affiliations:** 1 Military Hospital Ambala Cantt, Department of Pathology, Ambala Cantt, India; 2 Army College of Medical Sciences, Base Hospital Delhi Cantt, New Delhi, India; 3 Military Hospital Bareilly, Department of Pathology, Bareilly, India

**Keywords:** Creutzfeldt-Jakob Disease, Neuropathology, Prions, Electroencephalography, Autopsy

## Abstract

Sporadic Creutzfeldt-Jakob disease (CJD) is a rare neurodegenerative spongiform encephalopathy that causes neuronal derangement secondary to prion protein. Its initial diagnosis is often complex and challenging due to non-specific clinical presentation, lack of awareness, and low clinical suspicion. This disease is invariably fatal, and most patients die within 12 months of presentation. Definite diagnosis of prion disease requires neuropathological analysis, usually done at autopsy. Here, we present the autopsy findings of a 57-year-old male patient, illustrating the complexity of diagnosing this disease early in the clinical course and the need for a broad differential diagnosis at the onset.

## INTRODUCTION

Creutzfeldt-Jakob disease (CJD) is a sporadic disease with 100% fatality, which has an incidence of ~ 1 in 1 million and contributes to ~1 in 10,000 deaths. CJD is a prion disease that usually manifests in the 5th, 6^th^, or early 7th decade of life. Prions are proteinaceous substances with no genetic material. Prion diseases can manifest as infectious, genetic, or sporadic diseases, and no other group of illnesses with a single etiology can have such a wide range of manifestations.^1^ Most CJD forms are sporadic, accounting for 85-90% of cases, while the remaining forms are familial, iatrogenic, and variant forms. The most common clinical presentation includes rapidly progressive dementia, myoclonus, and ataxia. Additional signs are behavioral dysfunction, pyramidal/ extrapyramidal signs, and visual disturbances.^2^ Pre-mortem diagnosis of this entity is challenging due to its rarity, non-specific clinical presentation, and lack of awareness among clinicians, demanding a broad differential diagnosis at the onset. The common mimickers include other neurodegenerative diseases, autoimmune, infectious, vascular, and iatrogenic etiologies.^3^ Herein, we present an autopsy case of a 57-year-old male patient with a sporadic CJD. Diagnosis was established based on clinical presentation, neuroimaging (MRI), electroencephalogram (EEG), histopathological examination, and immunostaining for infected prion proteins on the brain autopsy.

## CASE REPORT

**Figure 1:**
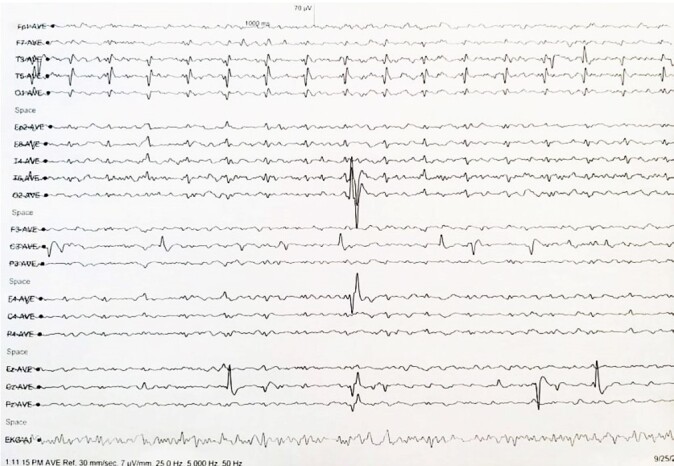
EEG features of continuous periodic biphasic and triphasic complexes and generalized slow waves.

A 57-year-old male presented with a history of focal left upper limb tremors of three weeks duration without any other localizing symptoms or signs and with no relevant history of similar complaints or any other medical illness. Clinical and laboratory examinations were normal. The brain and cervical spine magnetic resonance revealed focal cortical hyperintensity on the diffuse weighted image (DWI), with corresponding subtly appreciable hypointensity in the left precuneus on apparent diffusion coefficient (ADC) and FLAIR images but not on T2WI. The patient was managed symptomatically with an unremarkable course in the hospital, discharged, and kept on follow-up. A month later, he reported complaints of gradually progressive bilateral upper and lower limb weakness and pain, which was insidious in onset, and bilateral intentional tremors with worsening symptoms of progressive dyskinesias, increased tone, and hyperreflexia involving all four limbs. He was mildly disoriented and reported neuropsychiatric symptoms with hallucinations and insomnia. On examination, vitals were within normal limits. He exhibited a mask-like face, slurred speech, increased tone with cogwheel rigidity in all four limbs, myoclonic jerks, and dyskinesia. Power was grade 4-5 in all four limbs. Higher mental function and reflexes were normal. Dysdiadokokinesis was present, and the patient could not be ambulated due to pain and weakness. The patient was admitted as a case of Parkinson’s disease. On further evaluation, Electroencephalogram (EEG) showed periodic sharp wave discharges bilaterally throughout the EEG recording (Figure 1).

Slit-lamp ocular examination and nerve conduction studies were normal. Cerebrospinal fluid revealed albumin-cytological dissociation with increased protein and globulin but no cells. CSF bacterial and fungal cultures did not yield any pathogens and were negative for Mycobacteria by molecular diagnostics. Serum creatine phosphokinase, lactate dehydrogenase, and C-reactive proteins were raised. Serum anti-thyroid peroxidase was raised; however, all three thyroid hormones were normal. Serum Vitamin B12, Vitamin D, ceruloplasmin, N-methyl-D-aspartate (NMDA) receptor, α-amino-3-hydroxy-5-methyl-4-isoxazoleproprionic acid (AMPA) glucose transporter receptor 1, AMPA glucose transporter receptor 2, Gamma aminobenzoic acid (GABA)-B receptor antibodies, leucine-rich glioma inactivated-1 antibodies, contactin-associated protein-like 2 (CASPR2) antibodies were not contributory. 24-hour urinary copper was normal. The blood panel for autoimmune encephalitis was normal. A provisional diagnosis of Acute Immune Encephalitis was considered, and symptomatic management with broad-spectrum intravenous antibiotics and methylprednisolone pulse therapy was started.

**Figure 2:**
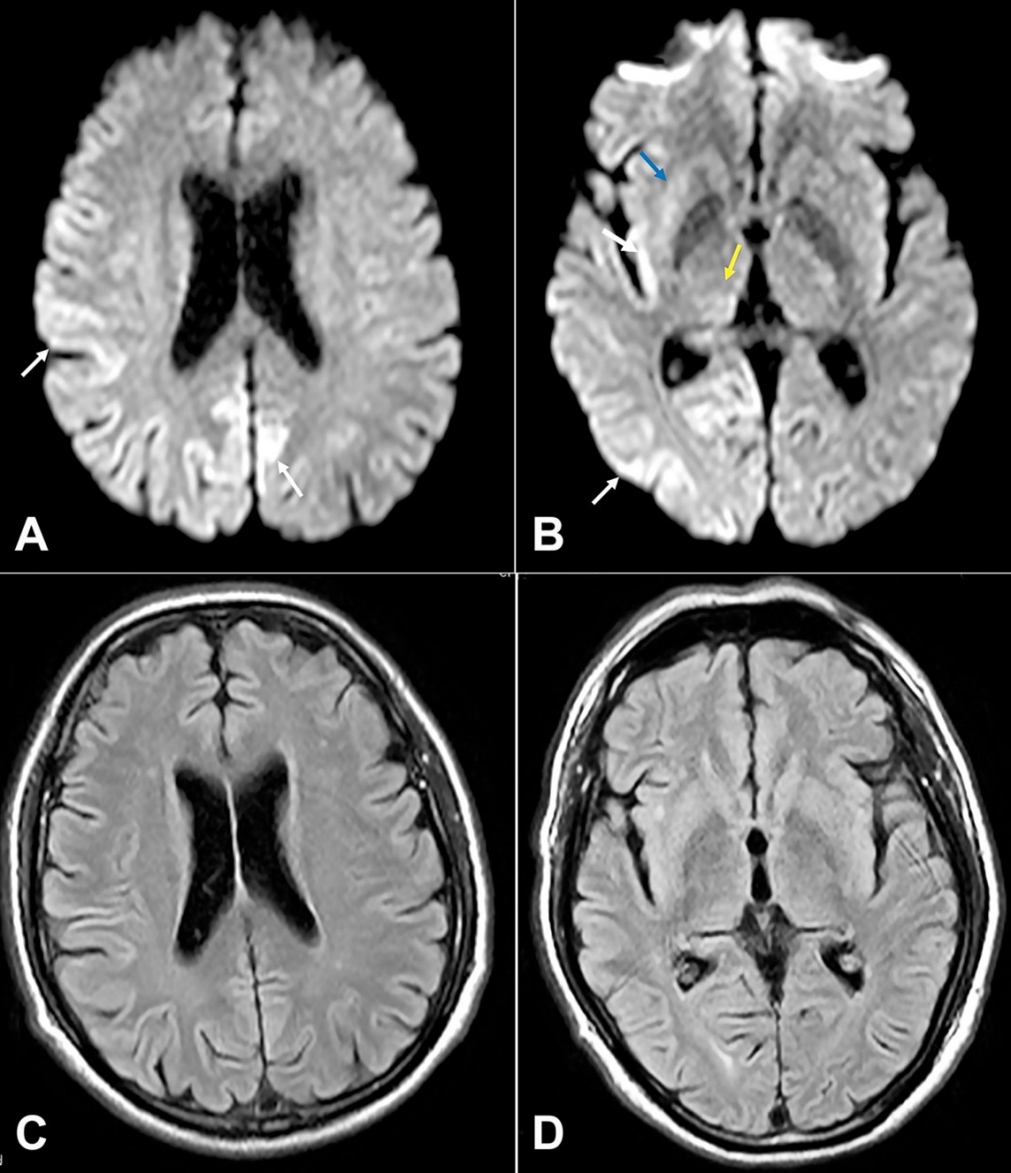
Brain MRI. **A –** DWI images (b 1000) at basal ganglia; and **B –** supraganglionic levels showing cortical hyperintensities (white arrows) in the frontal, parietal, occipital lobes and insular cortex, more predominant on the right side (the ‘Cortical Ribbon’ sign - white arrows) and also hyperintensity in the pulvinar of thalami (yellow arrow) bilaterally and the right putamen (blue arrow);**C** and **D –** FLAIR images at basal ganglia and supraganglionic levels show subtle hyperintensities in the cerebral cortex in the distribution described above and in bilateral pulvinar region. These findings are consistent with sporadic CJD.

Repeat magnetic resonance of brain and cervical spine after a week revealed widespread bilateral cortical hyperintensities on DWI with the corresponding hypointensity on ADC in an asymmetrical distribution, involving bilateral cingulate gyri, bilateral paracentral lobule, bilateral precuneus, bilateral angular and supramarginal gyri, bilateral occipital gyri, bilateral insula, bilateral inferior frontal gyri, and right inferior temporal gyrus. Changes were more widespread and prominent on the right side, with corresponding appreciable cortical FLAIR hyperintensity seen in most of these areas, representing the classical cortical ribbon sign typical of sporadic CJD (Figure 2).

The remaining basal ganglia appeared normal. The thalami, medial temporal lobes, and hippocampus were spared.

An impression of sporadic CJD was made. Subsequent EEG revealed periodic sharp wave discharges that further substantiated the diagnosis. The patient was administered symptomatic treatment with supportive end-of-life care, and the next of kin was counseled for a grave prognosis. The patient had a worsening in-hospital course and rapidly progressed to demise two months after his admission or three months after symptom onset.

Previous MRI images from the first imaging were reviewed retrospectively. Subtle cortical mild hyperintensity, less than that noted in the left precuneus, was noted in retrospect, but it was not considered abnormal or significant at that time in the absence of any significant clinical or neurological findings.

A limited autopsy comprising only brain retrieval was conducted, owing to the transmissible nature of Prion disease. No other organs were dissected for histopathological examination. As the patient presented at a remotely located tertiary care hospital in mountain terrain where advanced diagnostics were not available, hence the brain tissue fixed in 10% buffered formalin with triple packaging by United Nations guidelines for category B biological substance was sent to human brain tissue repository for neurobiological studies (HBTR), national research facility -department of neuropathology, National institute of mental health and neurosciences (NIMHNS) Bengaluru (India).

## AUTOPSY FINDINGS

A limited brain autopsy was performed using a disposable surgical gown, gloves, mask, and face shield protecting eyes and nose. All of the patient’s tissue was considered as being potentially infectious. Hence, post autopsy, all instruments and other objects potentially in contact were decontaminated by immersing in 1N NaOH for one hour, followed by autoclaving for at least 90 minutes at 134°C. Contaminated surfaces unsuitable for autoclaving were sponged down with 0.5% sodium hypochlorite solution after a contact time of two hours. ^4^ Representative sections from the frontal cortex and hippocampus were fixed in 96% formic acid for decontamination and processed for paraffin embedding to carry out histopathology and immunohistochemistry. Sections from all neuroanatomical areas were subsequently taken and processed for further evaluation.

On external examination, the corpse was averagely built and nourished, with a length of 169 cm and a weight of 78 kg. Puffiness of the skin, rigor mortis, and post-mortem lividity were present. Skin showed multiple venipuncture marks on the right and left arm.

**Figure 3:**
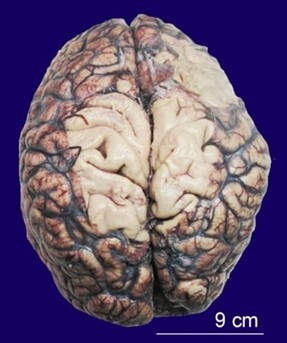
Gross view of the unfixed brain showing diffuse cortical atrophy of brain with meningeal congestion and thickening.

On gross examination of the brain, diffuse cortical atrophy with meningeal congestion and thickening was observed (Figure 3).

**Figure 4:**
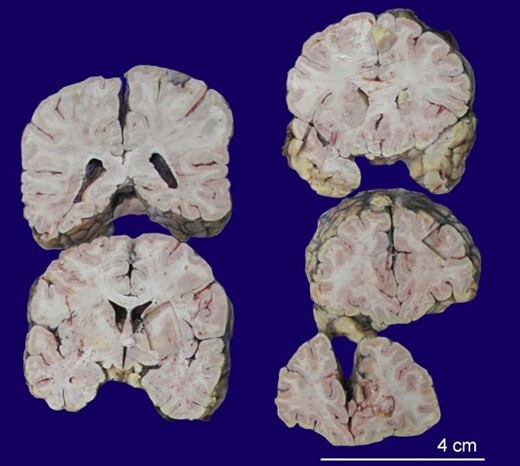
Gross view of the fixed brain serially sliced in coronal plane showing cerebral and cerebellar atrophy.

The cerebral hemispheres were sliced serially in the coronal plane, which revealed cerebral and cerebellar atrophy (Figure 4).

Atrophy was present in the frontal and parietal lobes, sparing the occipital lobes. Brain stem showed preserved pigmentation of Substantia Nigra.

**Figure 5:**
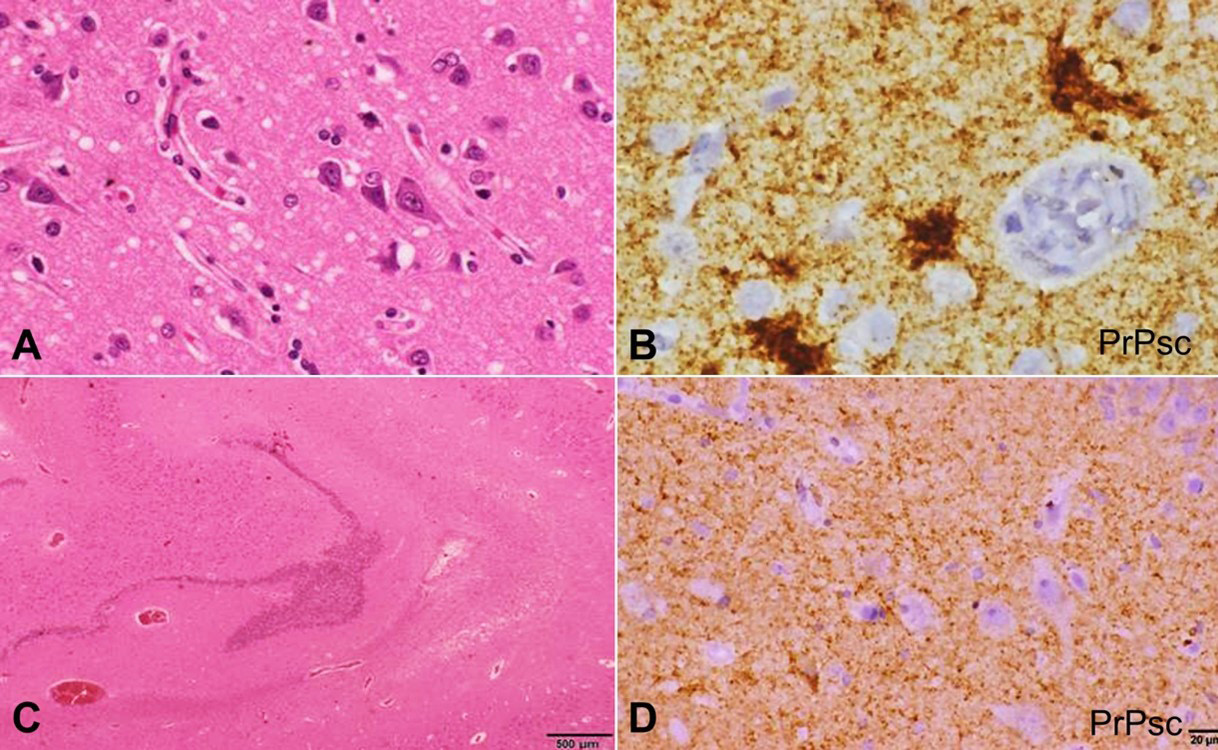
Photomicrographs of the Brain. **A –** Histopathological examination of frontal cortex show fine vacuolation of neuropil (H&E, 40X); **B –** Dense deposits of prion protein demonstrable in neuropil with formation of prion plaques (IHC for PrPsc, 40X); **C** and **D –** Sections from hippocampus also show diffuse deposits of PrPsc in a synaptic pattern (**C –** H&E, 10X; **D –** IHC for PrPsc, 40X).

Histopathological examination of the superior, frontal, dorsolateral, prefrontal, and orbitofrontal cortex showed diffuse cortical atrophy with significant neuronal loss. Spongiform changes were prominent in the superficial cortical layers, resembling a band-like layer below the molecular layer (Figure 5). Smaller vacuoles were noted, extending throughout the thickness of the cortex. White matter was relatively well preserved except for age-related leukariosis.

Immunohistochemistry performed with antibodies to PrPsc (clone KG9) along with positive control sections of confirmed CJD cases in the same batch showed intense deposition throughout the frontal cortex in a finely speculated synaptic pattern. In some places, perivacuolar deposits aggregating to form small plaques were seen. Plaques were more frequent at the depth of the sulci, correlating with MRI findings of "Cortical ribbon sign". The hippocampus revealed PrP plaques in the temporal cortex in addition to synaptic labeling patterns. Eventually, the final diagnosis of the sporadic form of CJD with unknown etiology was established at autopsy.

## DISCUSSION

CJD is a rapidly progressive neurodegenerative disease that occurs following hereditary or sporadic mutations of the prion protein-encoding gene.^2^ This disease could be acquired as genetic, infectious-like, iatrogenic, or variant CJD. Infectious cases are through the transmission of prion, and iatrogenic cases by corneal transplant, EEG and EMG electrode implantation, and dura mater grafts have also been reported.^5^ Variant CJD has been reported in young adults in Europe due to exposure to tainted beef from cattle with bovine spongiform encephalopathy.^6^ The genetic susceptibility to this disease is explained by the mutation of the PRNP gene located in the short arm of chromosome 20.^7^

CJD occurs sporadically in approximately 85% of patients, typically presenting in the sixth decade with no preference for sexes. sCJD occurs due to spontaneous transformation of prion protein or through somatic mutation.^8^

Based on the Centers for Disease Control and Prevention’s criteria for the diagnosis of sCJD, our patient had myoclonus, extrapyramidal signs (tremors, cogwheel rigidity) as clinical symptoms, hyperintensity signals in the caudate nucleus and/or putamen on diffusion-weighted imaging (DWI) or fluid-attenuated inversion recovery (FLAIR) MRI , and periodic sharp wave complexes on EEG, thus fulfilling the criteria of the “ probable” sporadic CJD.^9^ Although the definite diagnosis of sCJD can only be established by the detection of pathologic prion protein deposition in brain tissue, the diagnosis is supported by the detection of 14-3-3 protein in CSF, but due to limited resources, this immunoassay was not done in this case. 14-3-3 proteins are cytosolic polypeptides whose presence in CSF indicates general neuronal damage and, therefore, may be present in other conditions.^10^ Zerr et al.^11^ have demonstrated a sensitivity of 94% and specificity of 84% for 14-3-3 protein in the diagnosis of sCJD.

Neuroimaging by MRI is a valuable tool in CJD diagnosis, with bilateral areas of increased signal density affecting basal ganglia, FLAIR, and DWI being sensitive (91%) and specific ( 93%) for the entity.^12^ Periodic biphasic or triphasic sharp wave complexes on EEG of 1-2 Hz are prototypical in 90% of the cases. Steinhoff et al.^13^ studied 150 cases and reported a sensitivity of 64% and specificity of 91% of periodic complexes in diagnosing CJD. Variable unusual manifestations might lead to misdiagnosis; thus, MRI and EEG should be repeated during the follow-up period for confirmation, especially in patients with isolated visual or cerebellar symptoms.

The pathological changes of CJD would be confined to the Central Nervous System only, and the manifestation of any systemic abnormality like fever, leukocytosis, or raised ESR should alert the physician to search for an alternate diagnosis. No clinical blood tests are available for diagnosis or disease monitoring. The importance lies in the fact that before labeling a case as CJD, other treatable and manageable diagnoses like dementia with Lewi bodies, Alzheimer’s disease, movement disorders, Wilson’s disease, and autoimmune encephalitis should be excluded.^3^

Definite diagnosis for CJD depends on neuropathologic examination of the brain or autopsy. Autopsy with neuropathologic confirmation remains the only definitive way to make a diagnosis of CJD, which is challenging to perform due to various barriers, like fear of contamination of instruments, operation room, lack of adequately equipped mortuary, and proper training to handle such diseases.^14,15^

Moreover, it is a cause of concern among family members, clinicians, pathologists, intervention radiologists, and other healthcare workers in contact. ‘Virtopsy’ can be employed as an effective alternative to high-risk traditional autopsy procedures.^16^

CJD cases may have no discernible macroscopic changes, but many present with atrophy, spongiform degeneration, astrocytic gliosis, and amyloid plaques. Spongiform degeneration is characterized by many vacuoles in the neuropil between nerve cell bodies.^17^ Similar changes were confirmed on histopathology in this case also. These changes are usually found in the cerebral cortex, putamen, caudate nucleus, thalamus, and molecular layer of the cerebellum, hence the signs and symptoms.^18^

Hence the diagnosis of CJD mandates a correlation between clinical, epidemiologic, EEG, neuropathologic, and neuroimaging studies.^19^

## CONCLUSION

sCJD is a rare, invariably fatal, and rapidly progressing neurodegenerative human prion disease. It can mimic various other neurological disorders, and a high index of clinical suspicion, along with EEG, neuroimaging, and neuropathologic studies, is required to make a diagnosis. CJD may be suspected in a person in the 5th, 6^th^, or early 7th decade of life having dementia, myoclonus, and periodic electrical bursts and is afebrile. End-of-life care and adequate pain relief should be given to such patients.
